# Lateralization of Neural Speech Discrimination at Birth Is a Predictor for Later Language Development

**DOI:** 10.1111/desc.13609

**Published:** 2025-01-14

**Authors:** Lisa Bartha‐Doering, Vito Giordano, Sophie Mandl, Silvia Benavides‐Varela, Anna Weiskopf, Johannes Mader, Julia Andrejevic, Nadine Adrian, Lisa Emilia Ashmawy, Patrick Appel, Rainer Seidl, Stephan Doering, Angelika Berger, Johanna Alexopoulos

**Affiliations:** ^1^ Department of Pediatrics and Adolescent Medicine Comprehensive Center for Pediatrics Medical University of Vienna Vienna Austria; ^2^ Department of Developmental Psychology and Socialization University of Padova Padova Italy; ^3^ Austrian Institute of Technology Vienna Austria; ^4^ Department of Psychoanalysis and Psychotherapy Medical University of Vienna Vienna Austria

**Keywords:** fNIRS, language, language development, language lateralization, preterm birth, speech, speech discrimination

## Abstract

Newborns are able to neurally discriminate between speech and nonspeech right after birth. To date it remains unknown whether this early speech discrimination and the underlying neural language network is associated with later language development. Preterm‐born children are an interesting cohort to investigate this relationship, as previous studies have shown that preterm‐born neonates exhibit alterations of speech processing and have a greater risk of later language deficits. This investigation also holds clinical importance, as differences in neonatal speech discrimination and its functional networks may serve as predictors of later language outcomes. We therefore investigated neural speech discrimination using functional near‐infrared spectroscopy in 92 preterm‐ and term‐born neonates and its predictive value for language development in 45 of them. Three to five years later, preterm‐born and term‐born children did not significantly differ in language comprehension, sentence production, the use of morphological rules, or phonological short‐term memory. In addition, the gestational age at birth was not a significant predictor of language development. Neural speech discrimination, in contrast, was strongly correlated with later phonological short‐term memory. However, not the extent of speech discrimination, but rather its lateralization, was a predictor of language development. Children with less right hemisphere involvement—and therefore more left‐lateralized speech discrimination at birth—showed better development of phonological short‐term memory three to five years later. These findings suggest that the ability of fetuses to form memory traces is reflected by neonatal abilities to neurally discriminate speech, which in turn is a predictor for later phonological short‐term memory.

## 1 Introduction

The development of auditory areas in the brain already starts in the second trimester of pregnancy (Graven and Nrowne 2008; Kasprian et al. 2011). Their maturation is subsequently driven by both genetic factors and environmental stimuli (Chang and Merzenich 2003; Kienast et al. 2021; Le Guen et al. 2020; Vingerhoets et al. 2018). At birth, many newborns show neural mechanisms capable of detecting speech structure, thus neurally discriminating between speech and nonspeech (Gervain et al. 2008; Peña et al. 2003). When measured with functional near‐infrared spectroscopy (fNIRS), this neural capability is often reflected by both a quantitative difference of hemodynamic responses to speech versus nonspeech, and a specific neural localization of these speech‐specific responses (for review, see e.g., Januário et al. (2024), Quaresima, Bisconti, and Ferrari (2012)). While prosodic discrimination at birth has been shown to be predominantly processed in the right hemisphere (Martinez‐Alvarez, Benavides‐Varela, et al. 2023; Martinez‐Alvarez et al. 2023), phonetic‐phonological discrimination in term‐born neonates is often already lateralized toward the left hemisphere (Arimitsu et al. 2018). Several fNIRS studies in neonates using contrasting stimuli of forward and reverse speech have shown speech‐specific brain localization patterns, involving both bilateral temporal and frontal areas, with stronger activation observed in the left hemisphere compared to the right (Alexopoulos et al. 2021; Bartha‐Doering et al. 2019; Peña et al. 2003; Sato et al. 2012; Vannasing et al. 2016). This early lateralization of brain activations toward the left hemisphere thus seems to reflect an early specialization of language‐associated brain areas.

**Summary**

- Preterm‐born infants showed significantly less brain activity during speech discrimination at term‐equivalent age compared to term‐born neonates.
- Three to five years later, preterm‐ and term‐born children did not significantly differ in language abilities.
- The gestational age at birth was not a significant predictor of language development.
- In both preterm‐ and term‐born children, the lateralization of neural speech discrimination predicted later phonological short‐term memory.

However, not all neonates can neurally discriminate between speech and nonspeech. Previous work from our research group has shown that preterm‐born infants, for example, display significant differences in hemodynamic responses to forward versus reverse speech at term‐equivalent age when compared to term‐born infants (Bartha‐Doering et al. 2019). While preterm infants, as a group, do not show differences in hemodynamic responses to reversed speech compared to term‐born infants, they exhibit weaker hemodynamic responses to forward speech and show no significant difference between responses to forward and reversed speech. This indicates that they are not neurally capable of discriminating between these types of stimuli. These findings suggest that while preterm infants have typical basic acoustic and phonemic processing, they cannot neurally discriminate between forward and reversed speech. Children born before the gestational age of 32 weeks are especially vulnerable to speech versus nonspeech discrimination deficits at term (Alexopoulos et al. 2021). This significant delay has been associated with the lack of the very early, intrauterine auditory experience in preterm‐born infants (Bartha‐Doering et al. 2019; Monson et al. 2018). By the gestational age of 25 weeks, auditory stimuli from outside the womb reach the fetal auditory cortex, and auditory memory traces start to develop (Mahmoudzadeh et al. 2013; Partanen et al. 2013). Thus, while the fetus within the womb receives speech in utero low‐pass filtered by maternal tissue, preterm‐born neonates do not have this amount of auditory experience before delivery (Benavides‐Varela and Gervain 2017; Querleu et al. 1988). Neuroimaging studies in preterm‐born infants have shown delays in structural and micro‐structural brain maturation: diffusion tensor imaging studies reveal delayed maturation of the primary and secondary auditory cortex in preterms (Monson et al. 2018), and functional resting‐state studies in preterm‐born neonates exhibit less left lateralization of connectivity between fronto‐temporal brain areas at term‐equivalent age (Kwon et al. 2015). This decrease in functional connectivity within language networks in preterm‐born children can be observed throughout the following years of their lives (Choi et al. 2018). It can thus be hypothesized that the reduction of intrauterine auditory experience, coupled with the disruption of intrauterine development due to preterm birth, leads to a delay in the functional maturation of speech discrimination and the neural specialization of language‐related brain regions.

Already within the first years of life, preterm‐born infants show difficulties in speech perception more often than term‐born infants (Bosch 2011), and with 3 years of age, their expressive vocabulary is often less developed (Brosch‐Fohraheim et al. 2019). Preterm‐born children show lower levels and slower rates of language acquisition between 3 and 8 years of age (Landry, Smith, and Swank 2002). Research indicates that early language skills in preterm‐born infants are critical predictors of later language and academic outcomes (Guarini et al. 2010; van Noort‐van der Spek, Franken, and Weisglas‐Kuperus 2012). Studies have shown that deficits in early vocabulary and language processing can lead to persistent deficits in reading, verbal memory, and grammatical proficiency as children grow older (Vasilyeva et al. 2006). Cross‐sections neuroimaging studies in former preterm‐born individuals have shown an association of left language lateralization with language abilities: better language performance in former preterm‐born children and adults has been shown to be associated with a stronger functional lateralization of language processing (Murner‐Lavanchy et al. 2014; Stipdonk et al. 2022; Tseng et al. 2019), and better language abilities in preterm‐born adolescents have been associated with a stronger left lateralization of functional connectivity within the temporo‐parietal junction (Scheinost et al. 2015).

Nevertheless, fortunately, not all preterm‐born children experience language developmental deficits; in fact, more than half of those children show normal language skills, and many more show below average, but not reduced abilities (Stipdonk et al. 2018). Though prematurity is a risk factor for language developmental delay, gestational age at birth only explains about 35% of variance in verbal skills (Allotey et al. 2018). However, identifying children at risk for language developmental delay is important, as early intervention has been shown to be most effective (Fricke et al. 2013). Studies in older infants have shown an association of early auditory discrimination abilities and later language skills (Benasich and Tallal 2002; Kuhl and Rivera‐Gaxiola 2008). Phonological discrimination in 2‐ to 6‐month‐old infants is related to later literacy skills (Schaadt et al. 2015; van Zuijen et al. 2013), and prosodic discrimination in 6‐month‐old infants predicts vocabulary growth (Cristia and Seidl 2011). Thus, based on the previous findings, we hypothesized that both the extent of speech discrimination at birth and the underlying neural localization pattern have the potential to predict language development.

In the present study, we therefore investigated neonatal neural speech discrimination and later language development in a large group of preterm‐ and term‐born neonates. We used a fNIRS speech discrimination paradigm of forward versus reversed speech in neonates at term(‐equivalent age) and invited them back for language tests 3–5 years later. We explored whether the extent of differences in hemodynamic responses to speech versus nonspeech as well as their underlying neural localization is prognostic of later language development at an individual level. Based on our previous findings of neonatal speech discrimination deficits in preterm‐born infants (Alexopoulos et al. 2021; Bartha‐Doering et al. 2019), we hypothesized that the group of preterm‐born infants would show significantly less hemodynamic activations to speech forward stimuli compared to term‐born neonates, while their responses to reversed speech would be comparable. We thus hypothesized that preterm‐born neonates would exhibit less differences in their hemodynamic responses between forward and reversed speech compared to term‐born neonates. We furthermore hypothesized that preterms would show inferior language abilities 3–5 years later. Due to the reported heterogeneity in language outcome in former preterms, we furthermore hypothesized that there are only quantitative, but no qualitative, differences in the association of speech discrimination at birth and later language abilities between the groups of preterm and full‐term infants. Based on previous functional imaging studies in former preterm‐born children (Scheinost et al. 2015; Stipdonk et al. 2022), we furthermore hypothesized that less neural speech discrimination at birth would be associated with weaker language abilities at the age of three to five years.

## 2 Methods

### 2.1 Participants

Between 2015 and 2020, we prospectively included 115 neonates born at the Department of Pediatrics and Adolescent Medicine at the Medical University of Vienna. All infants met the following inclusion criteria: (1) normal auditory evaluation as measured by auditory brainstem response; (2) no neurological findings, that is, normal clinical examination and head ultrasound scan; (3) native German‐speaking parents; (4) normal language and reading development in both parents (according to their self‐report); and (5) no chromosomal or congenital anomalies. Some of these neonates were already participants in previous studies (Alexopoulos et al. 2022; Alexopoulos et al. 2021; Bartha‐Doering et al. 2019).

In all infants, fNIRS was performed. Term‐born infants (*n* = 46) were tested at birth, preterm‐born infants (*n* = 46) were tested at term‐equivalent age. After birth, most preterm‐born infants were nursed within the neonatal intensive care unit, but at the time of the fNIRS measurement, all former preterm‐born study participants were staying at the intermediate care unit. Study participants with motion artifacts in more than 50% of the trials in their fNIRS measurement and/or less than 18 remaining channels were excluded from further analyses. Twenty‐three measurements had to be excluded, resulting in fNIRS data from 92 infants.

**FIGURE 1:**
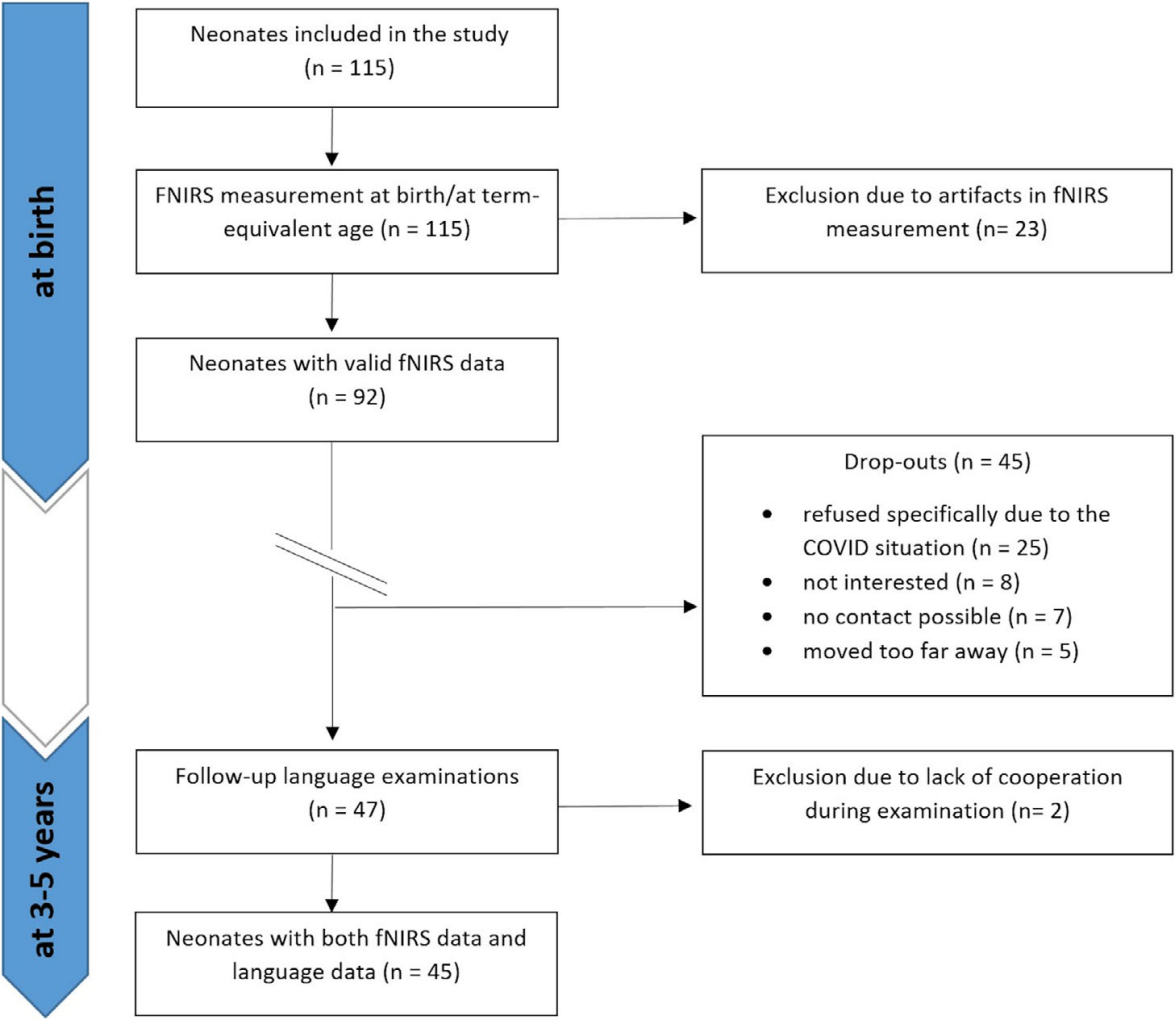
Participants’ inclusion and dropout from the study.

Between 2020 and 2023, follow‐up assessments were performed. We planned to re‐invite all children with valid fNIRS data (*n* = 92) at the age of 3 years to evaluate their language development. Due to the COVID‐19 pandemic, however, 25 parents refused to come to the hospital for the follow‐up investigation. Further dropout reasons are shown in Figure 1.

In the end, 45 participants were included in the final follow‐up analyses. Characteristics of the study group with valid fNIRS data (*n* = 92) and the final follow‐up group (*n* = 45) are shown in Table 1.

**TABLE 1:** Background information of study participants.

	Overall study group with valid fNIRS data (*n* = 92)		Study group with valid fNIRS and follow‐up data (*n* = 45)	
	Mean (SD)/median*	Range	Mean (SD)/median*	Range
Sex (f/m)	47/45		25/20	
**At birth**				
Preterm/full‐term	46/46		26/19	
Gestational age (weeks)	34.79 (5.36)	23.57–41.57	34.27 (5.61)	23.57–41.57
APGAR Score at 1 min (scale 1–10)	9	7–10	8	7–10
APGAR Score at 5 min (scale 1–10)	10	8–10	9	8–10
APGAR Score at 10 min (scale 1–10)	10	9–10	10	9–10
Weight (kg)	2.35 (1.11)	0.35–4.66	2.28 (1.21)	0.35–4.66
Height (cm)	45.24 (7.51)	28.00–56.00	44.38 (8.14)	28.00–56.00
Head circumference (cm)	31.19 (4.38)	21.00–38.00	30.78 (4.86)	21.00–38.00
**At fNIRS measurement**				
Gestational age (weeks)	38.34 (1.82)	36.29–41.72	38.10 (1.92)	36.29–41.71
Weight (kg)	2.90 (0.60)	1.88–4.66	2.89 (0.68)	2.00–4.66
Height (cm)	48.54 (3.72)	40.00–56.00	48.28 (3.77)	42.00–56.00
Head circumference (cm)	33.50 (1.86)	29.00–38.00	33.47 (2.10)	29.00–38.00
**At follow‐up investigation**				
Age (y)			3.77 (0.66)	3.12–5.69

*Note*: * mean is given in metric data, median in ordinal data. In all preterm children, the corrected age at follow‐up is shown (chronological age minus the number of weeks they were born too early).

The study was conducted in accordance with the Declaration of Helsinki (1973, revised in 1983) and approved by the Ethics Committee of the Medical University of Vienna. Written informed consent was obtained prior to the experiment from one parent or legal guardian per child.

### 2.2 FNIRS Measurement at Birth

The present study used methods similar to those in our prior fNIRS publications (Alexopoulos et al. 2022; Alexopoulos et al. 2021; Bartha‐Doering et al. 2019; Giordano et al. 2021). Consequently, some text included here is recycled from those sources.

#### 2.2.1 FNIRS Paradigm

We used a paradigm which has already shown robust findings in full term born neonates (Alexopoulos et al. 2022; Alexopoulos et al. 2021; Bartha‐Doering et al. 2019; Peña et al. 2003). This paradigm consists of forward and reverse speech samples of a female speaker reciting a children's story (Lobe and Weigel 1972). In the forward condition, 10 sequences of 15 s with well‐formed and complete prosodic units each (mean pitch 233 Hz) were presented. Each sequence comprised 2–4 German sentences. Mean intensity of sentences was equalized (mean intensity 70 dB). For the reverse condition, all sequences were time‐inverted using version 2.1.2 of Audacity(R) recording and editing software (Audacity 2012), thus generating reverse speech stimuli with the same acoustic and most phonetic features, but with distorted phonological, semantic, and prosodic information. Each sequence was followed by silence with randomized length (15–30 s). The order of sequences was pseudo‐randomized with no more than two consecutive sequences of the same condition, and counterbalanced across participants. The overall duration of the fNIRS paradigm was 9 min 10 s.

#### 2.2.2 FNIRS Data Acquisition

**FIGURE 2:**
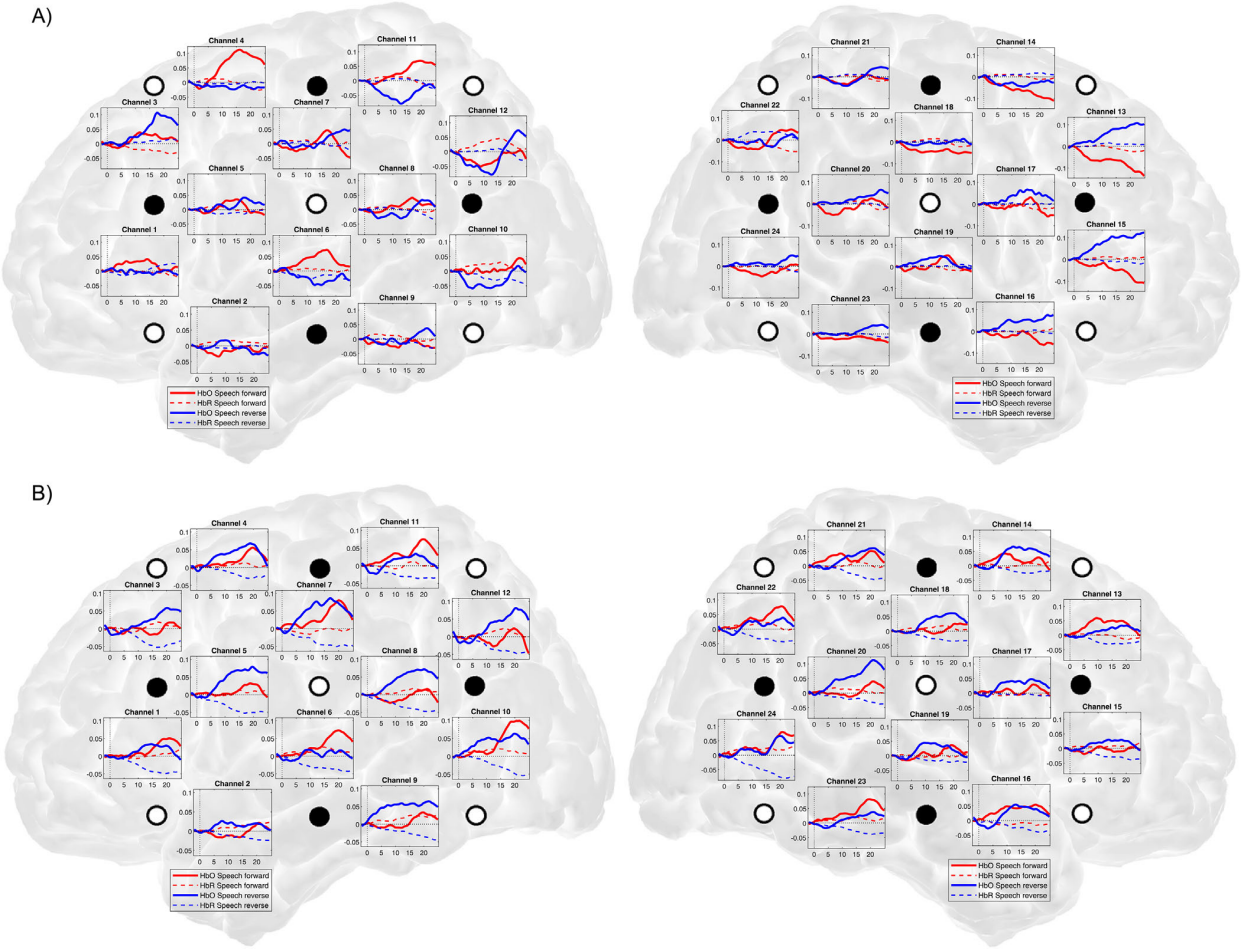
Mean time course of hemodynamic response for conditions speech forward (red) and speech reverse (blue) for each individual channel in term‐ (A) and preterm‐born neonates (B). Emitters are shown in light dots, detectors in black dots.

Hemodynamic responses were acquired using an ETG‐4000 optical topography system (Hitachi Medical Corporation, Japan) with 10 fibers for emission and 8 fibers for detection, resulting in a total of 24 channels. The sampling rate was set to 10 Hz, and the separation between each emitter and detector was 2 mm. The laser diodes emitted near‐infrared light with two different wavelengths at 695 and 830 nm, and total laser power set to 0.75 mW. The optical fibers were embedded in soft silicon cushions designed for use in neonates (Hitachi Neonate Probes). The optical fibers were placed on the head of the neonate and positioned directly above the ear, using the bilateral preauricular points as reference. The fNIRS probes were placed on the head using AtlasViewer software and according to the 10–20 system. They covered the inferior frontal gyri, the posterior part of the middle and superior frontal gyri, the inferior, middle and superior temporal gyri including the Sylvian fissure, the precentral and postcentral gyri, the supramarginal gyrus, and the inferior and superior parietal lobe. The probe design can be seen in Figure 2.

Infants were tested in a quiet, dimly lit room within the neonatal ward. During the fNIRS measurement, they were sleeping in their cribs. We only measured the hemodynamic responses when the neonates were in a state of regular (no body movements or startle reactions to stimuli, regular breathing) or irregular sleep (eyes closed but irregular breathing, muscle twitches, small body movements, or grimacing). In case the neonates woke up, the measurement was paused until their eyes were again closed and their breathing was even. One parent attended the measurement standing or sitting next to the crib. The position of the head was supported with a gauze diaper to ensure a straight posture of head and neck. The stimuli were presented using two loudspeakers positioned at a distance of approximately 2 m in front of the baby and at an angle of 30° from the infant's head.

#### 2.2.3 FNIRS Data Preprocessing

Preprocessing of fNIRS data was performed using open source software HOMER3 on MATLAB (R2019b, Mathworks, Natick, MA) (Huppert et al. 2009). For each participant, channels with a signal‐to‐noise ratio <2, likely due to non‐physiological noise, were discarded from further analysis. Moreover, only infants that had at least 50% of trials and at least 18 channels were included in the analysis. This led to the exclusion of 23 infants. In the remaining group, a mean of 23.75 channels (SD 0.82) (preterms mean 23.74, SD 1.00; term‐born mean 23.76, SD 0.60), and 99.98% of trials (SD 1.64) were included in the analyses (preterms mean 100, SD 0.00; term‐born mean 99.57, SD 2.32). Raw signals were then converted into optical density. Next, motion artifacts were automatically identified in each channel using the “hmR_MotionArtifactByChannel” function. Subsequently, two motion correction algorithms were applied (Di Lorenzo et al. 2019): (a) the Spline motion correction algorithm was used to correct for spike‐like motion artifacts previously identified and to correct for baseline changes due to step‐line artifacts, and (b) the wavelet motion correction technique was applied with an α parameter of 0.50 (Brigadoi et al. 2014; Molavi and Dumont 2012). Next, data were band‐pass filtered with cut‐off frequencies of 0.01 and 0.50 Hz to remove slow drifts and high frequency noise. Oxygenated hemoglobin (HbO) and deoxygenated hemoglobin (HbR) concentration changes were then computed using the modified Beer–Lambert law. For this, newborn‐appropriate differential path‐length factor (DPF) values were calculated using a general equation relating the DPF with age and wavelength: DPF (695 nm) = 5.31, DPF (830 nm) = 4.67 (Scholkmann and Wolf 2013). Baseline correction was performed by removing the mean signal of the 5 s preceding the stimulus. The hemodynamic response function (HRF) was extracted for each subject, channel, and condition by calculating the mean response from 2 s preceding the stimulus to 25 s post stimulus onset. Averaged HRF were subjected to further analysis. Since HbO is supposed to be the strongest indicator for neural responses in the neonatal fNIRS, (Gervain et al. 2011; Lloyd‐Fox, Blasi, and Elwell 2010), we report analyses of HbR changes, but further analyses were focused especially on HbO signal changes.

### 2.3 Evaluation of Language Development

In the follow‐up examination, language development was investigated using the “Sprachentwicklungstest für drei‐ bis fünfjährige Kinder” (SETK 3–5) (Grimm 2010). This well‐standardized test battery for children aged 3.0–5.9 years includes four age‐appropriate subtests within the categories of language comprehension, language production, and verbal short‐term memory. In the *Language comprehension* subtest, the child hears sentences of increasing syntactic complexity and length and has to move objects or point to one of four pictures. Language production abilities are investigated by two subtests: the subtest *Sentence production* asks the child to describe a picture showing an action, while the subtest *Use of morphological rules* tests the ability to form plural words. Finally, the subtest *Phonological short‐term memory* requires the child to repeat pseudowords with increasing phonological complexity and length. Standardized norms are available for all four subtests.

Raw scores of language subtests were transformed into age‐adjusted *z*‐scores. For preterm infants, their age at testing was first adjusted for prematurity by subtracting the number of weeks they were born early from their chronological age, and then compared to normative data. In line with clinical conventions, individual *z*‐scores from −1 to 1 were defined as average compared to normative data. Z‐scores above 1 were read as above average, *z*‐scores below −1 were read as below average compared to norms. ‐scores below −2 were interpreted as reduced.

### 2.4 Socioeconomic Status

At follow‐up, we furthermore performed a semi‐structured interview with one parent. Educational levels of the parents and the household income were used as indicators of the child's socioeconomic status (SES) (Bartha‐Doering et al. 2021). The educational levels of the parents were rated on a five‐point scale for the mother and the father, separately: (1) secondary school, (2) apprenticeship, (3) vocational school, (4) school leaving examination (general qualification for university entrance), and (5) university degree. Gross annual household income was classified on an eight‐point scale where higher scores reflect higher income, ranging from <10,000€, 10,000–19,000€, 20,000–29,000€, 30,000–39,000€, 40,000–49,000€, 50,000–59,000€, 60,000–69,000€, and above. The SES was calculated by taking the arithmetic mean of maternal education (5‐point scale), paternal education (5‐point scale), and household income (rescaled from 8‐point scale to 5‐point scale as: (income − 1)*4/7 + 1). Please see Bartha‐Doering et al. (2021) for more information.

### 2.5 Statistical Analyses

Statistical analyses were conducted using IBM SPSS Statistics (Version 28). Clinical background information, HbO, and HbR concentration changes were normally distributed as shown by the Kolmogorov–Smirnov Test for Normality (each *p* > 0.05). Thus, paired *t*‐tests, two sample *t*‐tests, and Pearson correlation were chosen in tests involving these data. Cognitive test results did not all follow a normal distribution, hence, nonparametric tests were chosen in analyses involving cognitive data. These statistical procedures included Mann–Whitney‐*U* Tests and Spearman correlations.

#### 2.5.1 FNIRS Data Analysis

To calculate the group mean hemodynamic response, we used a repeated measures ANOVA. The within‐subject factors were condition (speech forward/reverse) and hemisphere (left/right), and the between‐subjects factor was group (preterm/term). This analysis aimed to identify statistically significant differences in hemodynamic responses to both forward and reverse stimuli. Post‐hoc analyses were conducted using two‐sample and paired *t*‐tests, respectively.

The individual neural activity during speech discrimination (SP) was defined as the absolute difference in mean hemodynamic changes between conditions within all channels, and the individual neural activity during speech discrimination per hemisphere (SP_L_, SP_R_) was defined as the absolute difference in mean hemodynamic changes between conditions within all channels per hemisphere. We opted for using absolute values rather than algebraic ones, as half of our participants (*n* = 49/92) exhibited inverted HbO responses (i.e., negative HbO and positive HbR in response to stimuli). In group analyses, positive and inverted HbO responses would cancel each other out. Numerous prior fNIRS studies in neonates have reported inverted responses in some of their study participants (e.g., Abboub, Nazzi, and Gervain (2016); Issard and Gervain (2017); Sakatani et al. (1999); Telkemeyer et al. (2009)). Although possible causes for the relative decrease in oxygenation have been investigated, the underlying mechanisms behind these inverted responses remain unclear (see Issard and Gervain (2018), for a detailed discussion). In Supporting Information (Note 1), we additionally provide separate analyses for the group of participants exhibiting positive HbO responses and those with inverted HbO responses in order to be able to use the algebraic values of differences in mean HbO changes. Furthermore, we include all subsequent analyses for both groups separately.

A lateralization index of the neural activity during speech discrimination was then calculated in the overall brain by using the formula: LI_SP_ = ((SP_L _− SP_R_)/(SP_L _+ SP_R_))*100, with +100 representing complete left hemisphere dominance and −100 representing complete right hemisphere dominance of neural speech discrimination.

#### 2.5.2 Association of Neonatal Neural Activity During Speech Discrimination and Later Language Development

Spearman rank correlation was used to investigate the association of different parameters with cognitive test results. Significance is reported after Bonferroni correction for multiple testing (*p* < 0.05/number of variables). Variations of correlation slopes across groups were investigated by calculating univariate ANOVA fixed factor‐by‐covariate interaction. Differences in language test results between the groups of preterm‐ and term‐born children were calculated using the Mann–Whitney *U* Test.

## 3 Results

### 3.1 Neonatal Speech Discrimination in All Study Participants (*n* = 92)

#### 3.1.1 HbO

Repeated measures ANOVA in the overall group with valid fNIRS data (*n* = 92) revealed no significant within‐subject effect on HbO concentration changes for the factor condition (*F* (1, 90) = 0.61, *p* = 0.436, *η* ^2^_p_ = 0.007) nor hemisphere (*F* (1, 90) = 0.00, *p* = 0.947, *η* ^2^ *p* = 0.000), and no significant group effect on condition (*F* (1, 90) = 0.12, *p* = 0.732, *η* ^2^ *p* = 0.001) or hemisphere (*F* (1, 90) = 0.63, *p* = 0.429, *η* ^2^ *p* = 0.007), but a significant interaction of condition*hemisphere*groups (*F* (1, 90) = 5.63, *p* = 0.020, *η* ^2^ *p* = 0.059). Hence, the groups of preterm‐ and term‐born infants showed significantly different effects of the factors condition and hemisphere on HbO concentration changes. Mean time course of hemodynamic response for both conditions for each individual channel in term‐ and preterm‐born neonates are shown in Figure 2.

**FIGURE 3:**
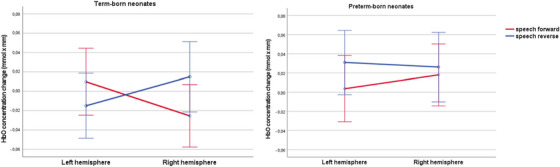
Interaction graphs (estimated marginal means) depicting the interaction effect between condition and hemisphere for each group. Error bars denote the standard error (±2).

Within the group of term‐born participants (*n* = 46), post‐hoc analyses revealed a significant interaction of hemisphere*condition (*F* (1, 45) = 4.58, *p* = 0.038, *η* ^2^ *p* = 0.092), with a significantly stronger HbO concentration change to forward speech in the left compared to the right hemisphere (*t* = 2.17, *p* = 0.018), and a stronger, though not significant, HbO concentration change to reverse speech in the right compared to the left hemisphere (*t* = −1.61, *p* = 0.058; Figure 3).

In the preterm group (*n* = 46), however, there was no significant effect of condition (*F* (1, 45) = 0.92, *p* = 0.342, *η* ^2^ *p* = 0.020) or hemisphere (*F* (1, 45) = 0.46, *p* = 0.500, *η* ^2^ *p* = 0.010), nor any interaction of condition*hemisphere (*F* (1, 45) = 1.08, *p* = 0.304, *η* ^2^ *p* = 0.023).

Preterm‐born neonates showed significantly reduced neural activity during speech discrimination compared to term‐born neonates across all channels (*t* = 2.21, *p* = 0.029; Table 2), as well as within each hemisphere—more pronounced in the left hemisphere than the right (SP_L_ *t* = 2.48, *p* = 0.008; SP_R_ *t* = 2.02, *p* = 0.047). The laterality index of neural speech discrimination LI_SP_, however, did not significantly differ between groups. Furthermore, individual neural activity during speech discrimination was not significantly associated with gestational age at birth, weight, height, or head circumference at birth, and also not with the socioeconomic background. Sex, however, had a significant impact on neural activity during speech discrimination: girls showed significantly higher neural activity during speech discrimination within the left hemisphere compared to boys (girls mean 0.16, SD 0.14; boys mean 0.11, SD 0.11; *t* = 2.01, *p* = 0.048).

**TABLE 2:** Individual neural speech perception after birth and language test results at follow‐up (*n* = 45).

	Mean (SD) [range]	Correlation with measures at birth				Sex differences* (p)	Correlation with SES r/r_s_ * (p)	Preterm‐born mean (SD) [range]	Term‐born mean (SD) [range]	Group differences* (p)
		Gestational age	Weight	Height	Head circumference					
**After birth (n = 92)**										
SP	0.12 (0.10) [0.00–0.40]	0.13 (0.229)	0.08 (0.434)	0.10 (0.364)	0.06 (0.565)	t = 1.84 (0.069)	0.21 (0.144)	0.10 (0.08) [0.00–0.38]	0.14 (0.11) [0.01–0.40]	**t = 2.21 (0.029)**
SP_L_	0.14 (0.12) [0.00–0.70]	0.13 (0.204)	0.09 (0.381)	0.11 (0.294)	0.11 (0.321)	**t = 2.01 (0.048)**	0.22 (0.163)	0.10 (0.09) [0.00–0.38]	0.17 (0.14) [0.02–0.70]	**t = 2.48 (0.008)**
SP_R_	0.13 (0.11) [0.00–0.46]	0.16 (0.129)	0.14 (0.189)	0.12 (0.275)	0.07 (0.488)	t = 1.02 (0.309)	−0.14 (0.361)	0.11 (0.09) [0.00–0.37]	0.15 (0.12) [0.02–0.46]	**t = 2.02 (0.047)**
LI_SP_	−0.77 (47.30) [−94.10–+87.11]	0.04 (0.689)	0.00 (0.993)	0.04 (0.723)	0.07 (0.511)	t = 1.03 (0.307)	0.20 (0.207)	−3.16 (47.54) [−94.10–+87.11]	1.61 (47.40) [−89.02–+79.20]	t = 0.48 (0.315)
**At follow‐up investigation (n = 45)**										
Language comprehension (*z*‐score)	0.38 (1.07) [−2.10–+2.20]	0.14 (0.372)	0.07 (0.647)	0.09 (0.543)	0.05 (0.765)	z = 0.54 (0.591)	0.14 (0.368)	0.31 (1.03) [−1.70–+2.20]	0.47 (1.15) [−2.10–+2.10]	z = 0.47 (0.637)
Sentence production (*z*‐score)	0.11 (1.05) [−2.70–+2.10]	**0.37 (0.025)**	**0.34 (0.042)**	0.28 (0.099)	0.18 (0.282)	z = 0.64 (0.537)	0.22 (0.185)	−0.21 (1.22) [−2.70–+2.10]	0.42 (0.76) [−1.20–+2.00]	z = 1.75 (0.081)
Use of morphological rules (*z*‐score)	−0.10 (1.24) [−2.10–+2.89]	0.11 (0.462)	0.12 (0.444)	0.15 (0.323)	0.15 (0.334)	z = 0.14 (0.891)	0.16 (0.292)	−0.25 (1.37) [−2.10–+2.89]	0.11 (1.04) [−2.00–+1.70]	z = 1.13 (0.259)
Phonological short‐term memory (*z*‐score)	−0.05 (1.24) [−2.89–+2.50]	−0.07 (0.673)	−0.08 (0.604)	−0.05 (0.749)	−0.06 (0.702)	z = 0.08 (0.936)	−0.04 (0.820)	0.07 (1.26) [−2.10–+2.10]	0.21 (1.23) [−2.89–+2.50]	z = 1.07 (0.283)

*Note*: SP, neural speech discrimination; SP_L_, neural speech discrimination in left hemisphere; SP_R_, neural speech discrimination in right hemisphere; LI_SP_, laterality index of neural speech discrimination; SES, socioeconomic background; * dependent on data distribution, parametric or nonparametric tests were chosen: *r*, Pearson correlation coefficient; *r*_s_, Spearman correlation coefficient; *t*, *t*‐value of two sample *t*‐test; *z*, *z*‐value of Mann–Whitney *U*‐test; *p* < 0.05 is indicated in bold letters.

#### 3.1.2 HbR

Repeated measures ANOVA in the overall group of participants with valid fNIRS data (*n* = 92) revealed no significant within‐subject effect on HbR concentration changes for the factor condition (*F* (1, 90) = 2.89, *p* = 0.093, *η* ^2^ *p* = 0.031) nor hemisphere (*F* (1, 90) = 1.58, *p* = 0.212, *η* ^2^ *p* = 0.017), no significant group effect on condition (*F* (1, 90) = 3.07, *p* = 0.083, *η* ^2^ *p* = 0.033) or hemisphere (*F* (1, 90) = 0.03, *p* = 0.868, *η* ^2^p = 0.000), and no significant interaction of condition*hemisphere*groups (*F* (1, 90) = 1.33, *p* = 0.252, *η* ^2^ *p* = 0.015). Hence, neither the whole group of participants nor the term‐born participants alone showed significant effects of the factors condition or hemisphere on HbR concentration changes. The following analyses therefore focused on HbO analyses.

Importantly, 49/92 neonates exhibited inverted hemodynamic responses, that is, negative HbO and positive HbR in response to stimuli. In group analyses, therefore, positive and negative values cancel each other out. In Supporting Information Note 1, we therefore report HbO analyses separately for the group with positive HbO values (group_pos_) and the group with inverted HbO responses (group_inv_).

### 3.2 Language Development (*n* = 45)

At the age of 3 years, children were invited back for a follow‐up investigation of language abilities. Five children were tested at the age of 5 years, as their follow‐up appointments had to be postponed for more than a year due to three COVID lockdowns. In these children, the language subtest *sentence production* was not administered, as no normative data are available for this age group. Test results and statistics excluding these children are shown in Supporting Information Note 2.

Overall, language test results were within or above the average range of normative data in 26 children, and below average (*z*‐score < −1) in 12 children. Seven children revealed reduced language functions (*z*‐score < −2 compared to norms) in one or more subtests of the language examination. More specifically, one child exhibited reduced language comprehension, two children showed reduced sentence production abilities, and one child performed below a *z*‐score of −2 in the subtest tapping morphological rules (four further children, however, did have a z‐score of exactly ‐2). Finally, phonological short‐term memory was reduced in six children.

The use of morphological rules and sentence production significantly correlated with each other (*r*_s_ = 0.520, *p* < 0.001), while language comprehension and phonological short‐term memory were not significantly associated with other language abilities. Furthermore, language test results were not significantly associated with height, weight, or head circumference at birth (Table 2). In addition, the language subtests did not significantly correlate with the children's SES and did not significantly differ by sex. Gestational age, however, correlated with the ability to produce sentences, but the significance did not survive correction for multiple comparisons (*r*_s_ = 0.37, *p* = 0.025). While the groups of preterm‐ (*n* = 26) and term‐born children (*n* = 19) did not significantly differ in their language abilities, there was a significant difference in the development of morphological rules within the subgroup of 3‐year‐olds (Supporting Information Note 2, Table S3).

### 3.3 Neonatal Speech Discrimination and Language Development (*n* = 45)

**FIGURE 4:**
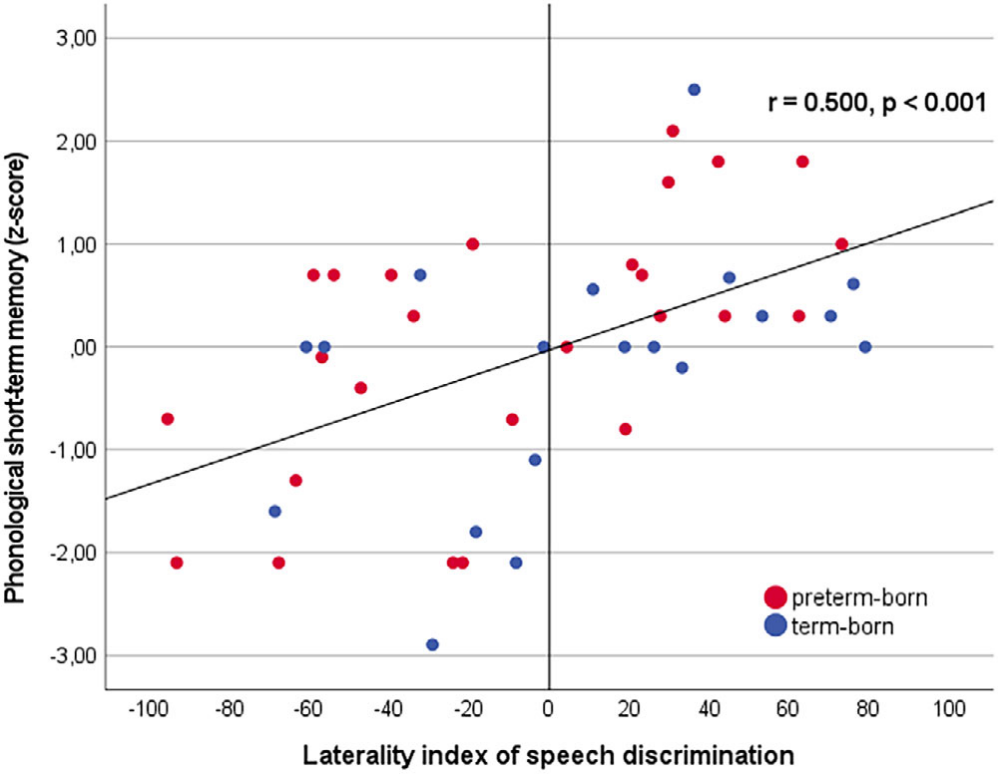
The laterality index of neonatal neural activity during speech discrimination significantly correlated with phonological short‐term memory 3–5 years later (*r*_s_ = 0.50, *p* < 0.001). The more left lateralized speech discrimination after birth, the better the development of phonological short‐term memory. All children with left lateralization of speech discrimination revealed average or above average phonological short‐term memory, whereas all children with below average (*z*‐score <−1, dotted red line) or reduced phonological short‐term memory (*z*‐score <−2, solid red line) exhibited right lateralization of speech discrimination.

Statistical analyses revealed that the extent of neonatal speech discrimination measured over all channels (SP) did not significantly correlate with language development 3–5 years later (Table 3). In addition, neural speech discrimination within the left hemisphere was not significantly associated with language comprehension, language production, the use of morphological rules, or phonological short‐term memory. However, neural speech discrimination within the right hemisphere was significantly negatively correlated with phonological short‐term memory (*r*_s_ = −0.57, *p* < 0.001). Hence, the less the absolute difference of hemoglobin changes between forward and backward speech within the right hemisphere, the better the development of phonological short‐term memory 3–5 years later. Consequently, the lateralization index of neural activity during speech discrimination LI_SP_ significantly correlated with the results of the phonological short‐term memory task (*r*_s_ = 0.50, *p* < 0.001; Figure 4). Hence, more leftward lateralization of speech discrimination at birth resulted in better phonological short‐term memory 3–5 years later. In contrast, language comprehension, use of morphological rules, and sentence production were not associated with lateralization of neural speech discrimination after birth.

**TABLE 3:** Correlation of neonatal speech discrimination and language development (*n* = 45).

	Correlation with neonatal speech discrimination *r*_s_ (*p*)			
	SP	SP_L_	SP_R_	LI_SP_
Language comprehension (*z*‐score)	−0.07 (0.665)	−0.14 (0.345)	0.00 (0.981)	−0.08 (0.591)
Sentence production (*z*‐score)	−0.22 (0.184)	0.30 (0.073)	−0.15 (0.364)	−0.11 (0.531)
Use of morphological rules (*z*‐score)	−0.02 (0.919)	−0.11 (0.475)	−0.08 (0.584)	−0.04 (0.819)
Phonological short‐term memory (*z*‐score)	−0.20 (0.199)	−0.03 (0.867)	−0.54 (<0.001)*	0.49 (<0.001)*

*Note*: SP, neural speech discrimination; SP_L_, neural speech discrimination in left hemisphere; SP_R_, neural speech discrimination in right hemisphere; LI_SP_, laterality index of neural speech discrimination; *p* < 0.05 is indicated in bold letters; * indicates significance after Bonferroni correction.

These findings remained stable when excluding all participants older than 3 years at follow‐up (Supporting Information Note 2, Table S4). Moreover, the significant association between neonatal speech discrimination and later phonological short‐term memory was present in both the preterm‐ and the term‐born group (preterm‐born group, *n* = 26: LI_SP_ × phonological working memory *r*_s_ = 0.62, *p* < 0.001; SP_R_ × phonological short‐term memory *r*_s_ = −0.57, *p* = 0.003; term‐born group, *n* = 19: LI_SP_ × phonological working memory *r*_s_ = 0.48, *p* = 0.039; SP_R_ × phonological short‐term memory *r*_s_ = −0.67, *p* = 0.002), and slopes of correlations between neonatal speech discrimination within the right hemisphere and later phonological working memory were not significantly different between the preterm‐ and term‐born group (group*LI_R_ *F* (1, 45) = 0.58, *p* = 0.453; group*LI_SP_ *F* (1, 45) = 0.16, *p* = 0.688).

## 4 Discussion

This longitudinal project investigated neural speech discrimination in 46 preterm‐born and 46 term‐born infants, and its predictive value for language development in a subgroup of 45 infants. As a group, preterm infants exhibited significantly less brain activity during speech discrimination at term‐equivalent age compared to term infants, and this difference was more prominent in the left than in the right hemisphere. These results replicate previous findings on speech discrimination deficits in preterm born children (Alexopoulos et al. 2021; Bartha‐Doering et al. 2019). Three to five years later, preterm‐ and term‐born children did not significantly differ in sentence production, sentence comprehension, the use of morphological rules, or phonological short‐term memory, and gestational age at birth was not a predictor of language development. Neonatal neural speech discrimination, however, predicted later phonological short‐term memory. However, not the extent of speech discrimination, but rather its lateralization toward the left hemisphere, was a strong predictor of language development.

### 4.1 Predictive Value of Right Hemisphere Involvement and Neural Lateralization of Speech Discrimination at Birth for Language Development

Cross‐sectional studies in older children have shown that atypical functional symmetries or even a rightward asymmetry of language‐related areas are associated with weaker language abilities in different pediatric populations, including specific language impairment, dyslexia, and autism spectrum disorder (de Guibert et al. 2011; Penolazzi et al. 2006; Whitehouse and Bishop 2008). In children born preterm, cross‐sectional studies similarly found more right hemisphere involvement and less language lateralization toward the left hemisphere being associated with weaker language abilities (Scheinost et al. 2015; Stipdonk et al. 2022). The predictive value of speech discrimination and its neural lateralization at birth for the development of language abilities, however, was unclear before the present study. This study is, to the best of our knowledge, the first longitudinal project on the association of neonatal language localization and later language development.

We examined speech discrimination at birth by employing a well‐known paradigm in which speech stimuli alternate with reversed speech (Alexopoulos et al. 2022; Alexopoulos et al. 2021; Bartha‐Doering et al. 2019; Bortfeld, Fava, and Boas 2009; Pena et al. 2003; Sato et al. 2012; Telkemeyer et al. 2009). These forward and reverse stimuli share the acoustic and most phonetic features, but differ in phonological, semantic, and prosodic information (Pellegrino et al. 2010). Assuming that the neonate is not yet able to process semantic information, differences in hemodynamic responses between these two kinds of stimuli reflect the neural capability to recognize phonetic/phonological and prosodic information. Prosodic information is primarily processed in the right hemisphere (Shapiro and Danly 1985). In contrast, phonetic and phonological processing is lateralized to the left hemisphere, involving regions such as the superior temporal sulcus, supramarginal gyrus, and angular gyrus (Minagawa‐Kawai, Cristia, and Dupoux 2011; Turkeltaub and Coslett 2010). These neural localizations for prosodic and phonetic/phonological processing have also been observed in term‐born neonates (Arimitsu et al. 2018; Martinez‐Alvarez, Benavides‐Varela, et al. 2023; Martinez‐Alvarez et al. 2023). Hence, an increase in left hemisphere involvement would reflect an increase in phonetic/phonological processing.

At birth, the language network is immature, displaying stronger interhemispheric and weaker intrahemispheric connectivity (Perani et al. 2011). With age, however, more focal language representations and an increase in the strength of left language lateralization can be seen (Arimitsu et al. 2011; Everts et al. 2009; Lidzba et al. 2011; Olulade et al. 2020; Scheinost et al. 2022; Szaflarski et al. 2012; Szaflarski et al. 2006). While the asymmetry in the infant's brain is not exclusive to language functions and is also observed within the sensory‐motor pathways of the corticospinal tract (Dubois et al. 2008; Dubois, Dehaene‐Lambertz, Soares, et al. 2008), the increased specialization of the left hemisphere for language processing during development aligns well with the results of the present study. The lateralization of neural speech discrimination in the present study was not driven by a significant increase of left‐sided activity, but by a significant decrease of right‐sided neural activity. We thus hypothesize that a reduction of bilateral speech discrimination and a more localized functional maturation of language‐associated areas within the left hemisphere is reflected by a stronger left lateralization of neonatal speech processing and is favorable for the further development of phonological short‐term memory. Consequently, we suggest that neonates who do exhibit left lateralization of speech discrimination at birth have already reached a developmental stage that facilitates further development. This hypothesis aligns with the findings of our previous study on structural brain asymmetry in fetuses, where a decrease of the right superior temporal sulcus asymmetry correlated with increased left language localization and better language abilities during childhood (Bartha‐Doering et al. 2023).

The investigation of speech discrimination and its neural lateralization at an individual level can help to explain the different findings in previous fNIRS studies in neonates. Most fNIRS studies in neonates reported a left lateralization of areas to already underlie early auditory speech discrimination (Alexopoulos et al. 2021; Bartha‐Doering et al. 2019; Peña et al. 2003; Vannasing et al. 2016); however, some studies found bilateral processing of auditory stimuli shortly after birth (Cabrera and Gervain 2020; Perani et al. 2011). These studies differ in the kind of stimuli used and in their fNIRS methodology and analyses; however, we suggest that these discrepancies could also be related to individual differences in brain maturation. In the present study, the involvement of the right hemisphere in neural speech discrimination negatively correlated with later language development. We suggest that, at an individual level, the lateralization of neural speech discrimination reflects the state of maturation of the functional neural language network in the neonate.

### 4.2 Neonatal Neural Speech Discrimination and Phonological Short‐Term Memory in Early Childhood

The significant association of neonatal neural speech discrimination and later phonological short‐term memory abilities found in the present study reflects both the anatomical and functional relationship of these cognitive capacities. The paradigm used in the present study consisted of forward and reversed speech stimuli. The time‐reversion created stimuli with the same acoustic and some phonetic features, but with distorted phonological, semantic, and prosodic information. We do not assume that semantic information is already accessible for neonates. Hence, the neural discrimination of forward versus reversed speech presumably relies on prosodic and/or phonological memory traces to (subconsciously) identify differences in these auditory stimuli (Boettcher‐Gandor and Ullsperger 1992; Cheour et al. 1998; Cheour et al. 1997). In the case of successful neonatal auditory discrimination of forward speech versus reversed speech, these memory representations must have been developed before birth. Indeed, the inner ear starts being functional at around 25 weeks of gestation (Lim and Brichta 2016; Mejdoubi et al. 2016). By this age, auditory input passes low‐pass filtered through the uterus and reaches the fetal auditory cortex, and auditory memory traces start to develop (Benavides‐Varela and Gervain 2017; Benavides‐Varela et al. 2012; Benavides‐Varela et al. 2017; Mahmoudzadeh et al. 2013; Partanen et al. 2013; Querleu et al. 1988). From this age on, imaging studies can detect neural activity in response to stimulation with different sound frequencies and faster fetal heart rates in reaction to language changes (Jardri et al. 2008; Minai et al. 2017). At the same time, an asymmetry in the structural changes of temporal brain areas starts to become visible, with a larger left‐sided temporal lobe and an earlier appearance of the right superior temporal sulcus (Bartha‐Doering et al. 2023; Kasprian et al. 2011). Hence, first auditory memory traces for speech appear to develop before birth, and storage and retrieval of these auditory representations are processed within the temporal lobes with an increasing lateralization toward the left hemisphere.

Phonological short‐term memory is the ability to briefly maintain and manipulate sounds important for speech and language. The most popular theoretical framework that conceptualizes phonological short‐term memory is Baddeley's theory of working memory (Baddeley 2003; Baddeley and Hitch 1974, 1986). One of its components is the phonological loop, which can hold memory traces for a few seconds and includes an articulatory rehearsal process. Retrieval and re‐articulation are used to refresh phonological memory traces, and word length and similarities between items influence the performance (Baddeley 2003). Deficits in phonological short‐term memory are associated with limitations in storage capacity and/or an atypical decay of items in memory. Studies have shown that the superior temporal gyri in both hemispheres, but especially in the left one, play a considerable role in phonological short‐term memory (Buchsbaum et al. 2005; Koenigs et al. 2011; Perrachione et al. 2017).

Thus, auditory speech discrimination and phonological short‐term memory both rely on the capacity to build and retrieve phonological memory traces, and they share underlying brain areas (Scott and Perrachione 2019; Zora, Schwarz, and Heldner 2015). Furthermore, both discrimination and phonological short‐term memory have been shown to be important for first (Benasich and Tallal 2002; Kuhl and Rivera‐Gaxiola 2008; Tsao et al. 2004) and second language learning (Ardila 2003; Garcia‐Sierra, Ramirez‐Esparza, and Kuhl 2016; Garcia‐Sierra et al. 2011; Juffs and Harrington 2011). Accordingly, studies in children with language developmental disorders have reported shortened durations of sensory memory traces, as measured with mismatch negativity, and reduced phonological short‐term memory (Kujala and Leminen 2017; Norrelgen, Lacerda, and Forssberg 1999). Based on the findings of our longitudinal study, we thus hypothesize that the ability of fetuses to form phonological memory traces is reflected by neonatal speech discrimination abilities, which is a precursor of phonological short‐term memory in early childhood.

### 4.3 What About the Other Language Abilities?

Language comprehension, sentence production, and the use of morphological rules, in contrast, were not predicted by neonatal speech discrimination. There might be several reasons for this lack of association. First, the age at follow‐up testing may have been too young to reliably measure these skills, as sentence production and the use of morphological rules just start to develop at the end of the second or beginning of the third year of life (Rispoli 2019). Phonological short‐term memory, in contrast, already starts to develop in the first 12 months (Nelson 1995). The language tests used in the present study may thus lack sensitivity for this young age range, whereas the language test tapping phonological short‐term memory might have had a better standardization for this age group. Second, phonological short‐term memory was the most commonly impaired cognitive ability within the follow‐up group, which might have increased the possibility to detect significant associations. Third, pseudoword repetition might be a more defined, circumscribed measure compared to other language tests. While a cognitive test of repeating pseudowords is also influenced by general attentional abilities, auditory processing, and speech‐motor abilities, it has been shown to primarily measure phonological working memory in a vast amount of pediatric studies over the last decades (see e.g., Archibald and Gathercole (2006), Gathercole (2006), Gathercole and Alloway (2006)). Moreover, large studies in search of genes modulating language abilities in developmental language disorders often found nonword repetition to be linked to specific genetic loci (Newbury, Bishop, and Monaco 2005; Newbury et al. 2009). Besides providing genetic evidence for the importance of phonological short‐term memory in language acquisition, these findings point to the possibility that other language tests, including language comprehension and syntactic processing, might not tap such a circumscribed, specific developmental function. Fourth, the results of the present study may reflect the fact that the other language abilities are less linked to the auditory cortex compared to phonological short‐term memory. As auditory speech discrimination is predominantly processed within the temporal brain areas, earlier regional maturation processes might be better reflected by subsequent phonological short‐term memory abilities.

Phonological short‐term memory per se has not only been shown to identify developmental language disorders (Archibald and Gathercole 2006, 2007; Archibald 2018; Schwob et al. 2021), but also to have an important influence on subsequent language learning (Baddeley, Gathercole, and Papagno 1998; Delcenserie et al. 2021; Gathercole et al. 1992; Pierce et al. 2017). However, within the 3‐ to 5‐year‐old study participants, the present study did not reveal any association of the children's phonological short‐term memory with language comprehension, the use of morphological rules, or sentence production. This might again be due to the early developmental period at follow‐up testing, which might not have fully covered the language developmental capacities of the children. At these early ages, phonological working memory might be primarily related to tests on vocabulary knowledge (Gathercole and Adams 1994; Gathercole and Baddeley 1993). Unfortunately, we did not test vocabulary in our children. This is a limitation of our study, especially considering that previous research has demonstrated that preterm‐born children often exhibit a less developed expressive vocabulary (Brosch‐Fohraheim et al. 2019). It may, however, also be that the pseudoword repetition task administered in the present study tapped auditory memory functions independent of the development of other language abilities. Hence, at present we cannot confirm that the phonological short‐term memory findings actually predict further language development in our study participants. To clarify this question, longer follow‐up periods are needed.

### 4.4 The Influence of Preterm Birth on Neonatal Speech Discrimination and Language Development

In the present study, preterm‐born and term‐born infants showed significantly different interaction effects of the factors condition and hemisphere on HbO concentration changes. Differences in speech discimination in preterms compared to term‐borns were already reported in previous studies (Alexopoulos et al. 2021; Bartha‐Doering et al. 2019). These studies did, however, not observe differences in hemispheric responses or interaction effects. The current study's data indicate that differences in neural speech discrimination between preterm and term‐born infants are greater in the left hemisphere than in the right. Additionally, the mean laterality index tends to be more rightward in the preterm group, but this difference is not statistically significant, and the range of laterality indices is wider in the preterm group. It can be hypothesized that the larger sample size in the present study (*n* = 92), compared to previous studies, may help detect smaller effects. However, it is possible that even larger sample sizes are needed to observe hemispheric differences between preterm and term‐born infants. Alternatively, the preterm group may consist of a more heterogeneous sample of neonates, which could also influence the results.

Contrary to our hypotheses, our study found few differences in language development between preterm‐ and term‐born children. The group of preterm‐born children showed lower means in all language tests, but between‐group statistics did not reach significance in any language test. Furthermore, gestational age at birth was not significantly correlated with later language abilities. This might be due to our relatively small study group, and studies including larger sample sizes often describe significant differences in various language skills in preterm‐born compared to term‐born children (Allotey et al. 2018). Our study, however, suggests that preterm birth per se does not imply language developmental delays; many preterm‐born children in our study performed within the normal range of developmental language tests.

### 4.5 Inverted HbO Responses

More than half of our participants exhibited inverted HbO responses, that is, a decrease of HbO in response to stimuli. Numerous prior fNIRS studies in neonates have reported inverted responses in some of their study participants (Abboub, Nazzi, and Gervain 2016; Issard and Gervain 2017; Sakatani et al. 1999; Telkemeyer et al. 2009). This phenomenon is particularly common in neonates and young infants, where inverted HbO responses have been observed especially in the temporal cortex in response to both speech and nonspeech sounds. Interestingly, Arimitsu et al. (2018) demonstrated that preterm‐born infants tested shortly after birth exhibit this inverted response more frequently, with gestational age at birth being associated with this atypical response pattern. In the present study, we also found inverted HbO more often in preterm‐ than in term‐born infants, although this finding was not statistically significant. However, inverted responses have also been observed in term‐born neonates, as well as across participants within the same condition (Sakatani et al. 1999), within participants across different conditions, and within the same age group or brain region (Abboub, Nazzi, and Gervain 2016; Issard and Gervain 2017; Telkemeyer et al. 2009). Although possible causes for the relative decrease in oxygenation have been investigated, the underlying mechanisms behind these inverted responses remain unclear to date (see Issard and Gervain (2018), for a detailed discussion). What is noteworthy, is that in our study, group_pos_ and group_inv_ showed significant differences in their Apgar scores after birth. Although this difference was small and only evident in the Apgar score at 10 minutes, not in the earlier assessments, it may provide a clue to the underlying cause of the inverted HbO responses. The Apgar score is a rapid assessment tool used to evaluate a newborn's physical condition based on appearance, pulse, grimace, activity, and respiration. A lower Apgar score at 10 min can indicate issues such as prematurity, oxygen deprivation, infection, neurological problems, or metabolic disorders. Although we only included neonates with normal neurological findings and without chromosomal or congenital anomalies, it is possible that variations in oxygen supply, respiration, or metabolic functions contributed to differences in brain hemodynamics, potentially reflected in the inverted HbO responses. Further research, however, is needed to explore more on the underlying causes of inverted HbO responses.

Due to these inverted responses in some of our study participants, we chose to use the absolute differences between conditions for the correlation analyses related to speech discrimination, as algebraic values would counteract one another in group analyses. In the Supporting Information, however, we show an alternative approach to this problem: we divided our participants in two groups according to the direction of their HbO response (group_pos_ and group_inv_), used the algebraic values of differences between conditions, and run the subsequent analyses separately per group. This method revealed statistical differences in mean HbO changes across different conditions much more clearly. It furthermore yielded comparable results with regard to the association of speech discrimination at birth and language development by showing a decrease of right hemisphere speech discrimination being associated with better phonological short‐term memory. This approach of dividing group according to the direction of their HbO response is not very common in fNIRS research. Although this method does not allow for a calculation of laterality and requires a larger number of study participants to form two groups, it may aid in detecting hemodynamic differences between conditions more easily in future neonatal fNIRS studies.

### 4.6 Limitations

The present study focused on the neural lateralization of speech discrimination, using a block design approach to investigate differences between conditions and hemispheres. The blocks in this study comprised two to four sentences each, alternating between forward and reverse presentation. While block designs possess the highest signal‐to‐noise ratio, statistical power, and maximal time efficiency and are therefore the most common experimental design to use in fNIRS experiments (Issard and Gervain 2018; Luke et al. 2021), they do not provide information about the time course of the hemodynamic response to a single event, such as a single sentence. Future fNIRS studies interested in the time course of sentence processing may therefore consider event‐related designs where each sentence is presented individually.

It can be hypothesized that additional factors may influence the development of language‐specific neural networks, including early parent‐child interaction (Caskey et al. 2014). This interaction is particularly challenging in cases of preterm birth, where neonates are often cared for in the NICU and incubators, conditions known to significantly affect language development in preterm neonates (Bertsch et al. 2020; Pineda et al. 2014). Unfortunately, we did not collect data on the duration or quality of parent‐child interaction. Future studies on the early development of language‐associated brain areas should consider incorporating measures of parent–child interaction into their study design.

## 5 Conclusion

This study suggests that neural speech discrimination at birth is a predictor for the development of phonological short‐term memory in early childhood. Specifically, we were able to demonstrate a significant association between neonatal language localization and further phonological short‐term memory. These results underline the importance of pre‐ and early postnatal hearing and learning, and provide new information about the very early developmental trajectories of auditory language learning.

## Conflicts of Interest

The authors declare no conflicts of interest.

## Ethics Statement

The study was conducted in accordance with the Declaration of Helsinki (1973, revised in 1983) and approved by the Ethics Committee of the Medical University of Vienna (Nr. 1215/2014). Written informed consent was obtained prior to the experiment from one parent in all children.

## Supporting information



Supporting Information

## Data Availability

Data of the study are available at https://osf.io/2pvdm/?view_only=dba0f3da474f402ab1d168e090dcedcc.
