# Supplementary material for: Lateralization of Neural Speech Discrimination at Birth Is a Predictor for Later Language Development
Source: Dev Sci. 2025 Jan 14;28(2):e13609. doi: 10.1111/desc.13609 (PMC11730390; doi:10.1111/desc.13609)
Supplement: Supplementary file 1 — Supporting Information [file DESC-28-e13609-s001.docx]

**Supplementary file**

**Supplementary Note 1**

More than half of our participants (n = 49/92) exhibited inverted HbO in response to stimuli, i.e. negative HbO and positive HbR in resonse to stimuli. In the main manuscript, we chose to use absolute values instead of algebraic ones for correlation analyses between neural discrimination and later language development. This approach was taken because, in group analyses, positive and inverted HbO responses would cancel each other out. However, here we additionally provide separate analyses of the relative differences in mean HbO changes for the group of participants exhibiting positive HbO responses (group_pos_) and those with inverted HbO responses (group_inv_).

For each group, we separately performed a repeated measures ANOVA with within-subject factors condition (speech forward/reverse) and hemisphere (left/right), and between-subjects factor group (preterm/term). In addition, post-hoc analyses were conducted using two-sample and paired t-tests, respectively, within group_pos_ and group_inv_, respectively. The individual neural activity during speech discrimination (Spa) and speech discrimination per hemisphere (SPa_L_, SPa_R_) was defined as the **algebraic** difference in mean HbO changes between conditions within all channels per hemisphere. A calculation of the laterality index, however, is not possible due to negative values.

*Differences between Group_pos_ and Group_inv_*

In group_pos_, there were more fullterm-born than preterm-born neonates, while in group_neg_, preterm-born neonates were more prevalent (Table S1). Group_pos_ and the group_inv_, however, did not significantly differ in preterm birth. Furthermore, they did not differ in sex, gestational age, head circumference, weight, and height at birth or at measurement (all p > 0.05). However, the groups significantly differed in their APGAR Scores at 10 minutes. Per definition, the groups also significantly differed in their speech discrimination (all p < 0.001).

Table S1. Background information, speech discrimination, and differences between group_pos_ and group_inv_

|  | **Study group with positive HbO values (n = 43)** | | **Study group with inverted HbO values (n = 49)** | | **Group differences** |
| --- | --- | --- | --- | --- | --- |
|  | **Mean (SD)/ median*** | **Range** | **Mean (SD)/ median*** | **Range** | **(p)**** |
| **Background information** |  |  |  |  |  |
| Sex (f/m) | 23/20 | | 24/25 | | z =-0.43 (0.668) |
| **At birth** |  |  |  |  |  |
| Preterm / full term | 19/24 | | 27/22 | | z = -1.04 (0.299) |
| Gestational age (weeks) | 35.75 (4.60) | 25.86 – 41.29 | 33.97 (5.86) | 23.57 – 41.57 | t = 1.63 (0.106) |
| APGAR Score at 1 min (scale 1-10) | 9 | 7 – 10 | 8 | 7 – 10 | z = -1.52 (0.129) |
| APGAR Score at 5 min (scale 1-10) | 10 | 8 – 10 | 10 | 8 – 10 | z = -1.36 (0.175) |
| APGAR Score at 10 min (scale 1-10) | 10 | 9 – 10 | 10 | 9 – 10 | **z = -2.29 (0.022)** |
| Weight (kg) | 2.50 (1.01) | 0.35– 3.94 | 2.22 (1.18) | 0.57– 4.67 | 1.22 (0.225) |
| Height (cm) | 46.45 (6.49) | 31.45 – 56.00 | 44.17 (8.22) | 28.00 – 56.00 | 1.48 (0.141) |
| Head circumference (cm) | 32.10 (3.66) | 22.00 – 38.00 | 30.40 (4.82) | 21.00 – 38.00 | 1.92 (0.059) |
| **At fNIRS measurement** |  |  |  |  |  |
| Gestational age (weeks) | 38.22 (1.77) | 34.29 – 41.43 | 38.44 1.87) | 35.00 – 41.72 | 0.58 (0.566) |
| Weight (kg) | 2.88 (0.56) | 1.88 – 3.94 | 2.92 (0.64) | 1.90 – 4.66 | 0.32 (0.753) |
| Height (cm) | 48.69 (3.62) | 40.00 – 56.00 | 48.41 (3.83) | 41.50 – 56.00 | 0.36 (0.723) |
| Head circumference (cm) | 33.67 (1.61) | 30.00 – 38.00 | 33.35 (2.06) | 29.00 – 38.00 | 0.81 (0.422) |
|  |  |  |  |  |  |
| **Speech discrimination** |  |  |  |  |  |
| SPa | 0.11 (0.10) | 0.00 – 0.40 | -0.12 (0.10) | -0.01 – -0.38 | **11.54 (0.001)** |
| SPa_L_ | 0.13 (0.16) | -0.11 – 0.71 | -0.15 (0.12) | -0.44 – 0.09 | **8.50 (<0.001)** |
| SPa_R_ | 0.10 (0.11) | -0.09 – 0.43 | -0.31 (0.14) | -0.46 – 0.21 | **8.79 (<0.001)** |

Note. * In ordinal data, the median is given; ** dependent on the data type, two sample t-test or Mann-Whitney-U Test was performed

*Neonatal Speech Discrimination in Group_pos_ (n = 43)*

Repeated measures ANOVA in group_pos_ revealed a significant within-subject effect on HbO concentration changes for the factor condition (F (1,41) = 56.35, p < 0.001, η^2^p = 0.579), and post-hoc paired t-test revealed significantly higher HbO mean values in response to forward compared to reverse stimuli (forward mean = 0.06, SD 0.09, reverse mean = -0.05, SD 0.11; t = 7.66, p < 0.001). Figure S1 depicts the mean time course of hemodynamic response for both conditions within each individual channel, as well as a channel-wise paired t-test comparison between the mean HbO of conditions.

ANOVA, however, did not show a significant within-subject effect of hemisphere (F (1,41) = 1.01, p = 0.321, η^2^p = 0.024). Furthermore, no significant group effect on condition (F (1,41) = 0.12, p = 0.278, η^2^p = 0.029) or hemisphere (F (1,41) = 1.47, p = 0.233, η^2^p = 0.034), and no significant interaction of condition*hemisphere*groups (F(1,41) = 2.61, p = 0.114, η^2^p = 0.060) was observed.

Hence, within group_pos_, neonates revealed significantly different HbO responses to speech forward compared to backward. Furthermore, preterm-born and term-born infants showed comparable effects of the factors condition and hemisphere on HbO concentration changes.

*
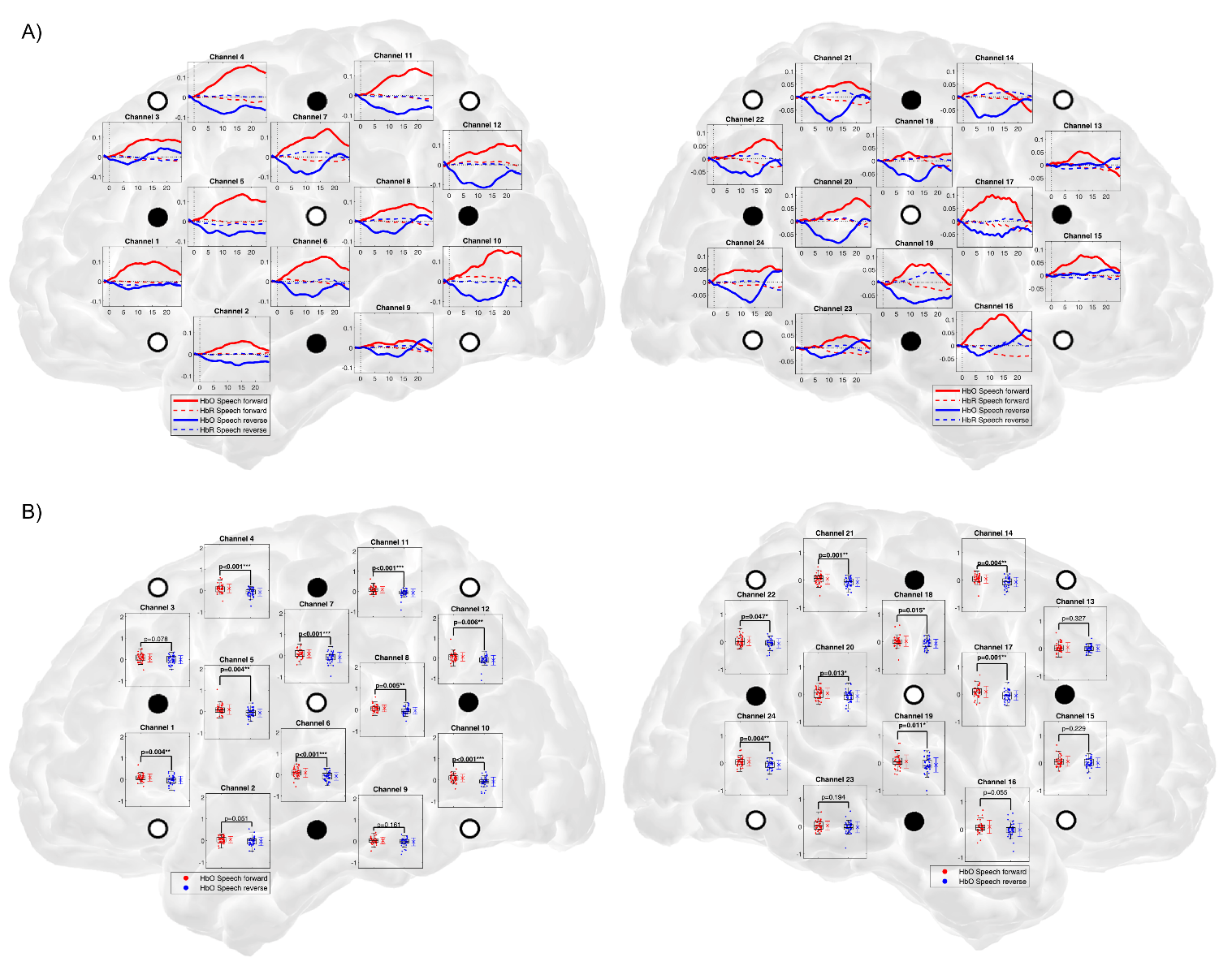
*Figure S1. Group_pos_ A) Mean time course of hemodynamic response for forward speech (red) and revese speech (blue) for each individual channel; B) channel-wise paired t-test comparison of mean HbO between conditions.

*Neonatal Speech Discrimination in Group_neg_ (n = 49)*

In group_neg_, repeated measures ANOVA again revealed a significant within-subject effect on HbO concentration changes for the factor condition (F (1,47) = 85.13, p <0.001, η^2^p = 0.644), and a significant group effect on condition (F (1,47) = 4.27, p = 0.044, η^2^p = 0.083). Post-hoc paired t-test showed significantly lower (i.e., more negative) HbO mean values in response to forward compared to reverse stimuli (forward mean = -0.05, SD 0.08, reverse mean = 0.07, SD 0.07; t = 6.78, p < 0.001) in the overall group (Figure S2). Two-sample t-test furthermore exhibited significantly lower (i.e., more negative) HbO mean values in response to forward speech in term-born compared to preterm-born neonates (term-borns mean = -0.08, SD 0.08; preterm-born mean -0.03, SD 0.07; t = 2.20, p = 0.033), but not significantly different HbO mean values in response to reverse speech (fullterm mean 0.08, SD 0.06; preterm-born mean 0.07, SD 0.06; t =0.43, p = 0.673).

Again, no significant within-subject effect of hemisphere (F (1,47) = 1.82, p = 0.184, η^2^p = 0.037) was found, and no significant interaction of condition*hemisphere*groups (F(1,47) = 2.70, p = 0.107, η^2^p = 0.054) was observed.

In sum, also in group_neg_ significantly different HbO responses to speech forward compared to reverse speech were found. Furthermore, preterm-born and term-born infants showed significantly different HbO concentration changes to speech forward, while their HbO responses to reverse speech were comparable to the full-term group.

*
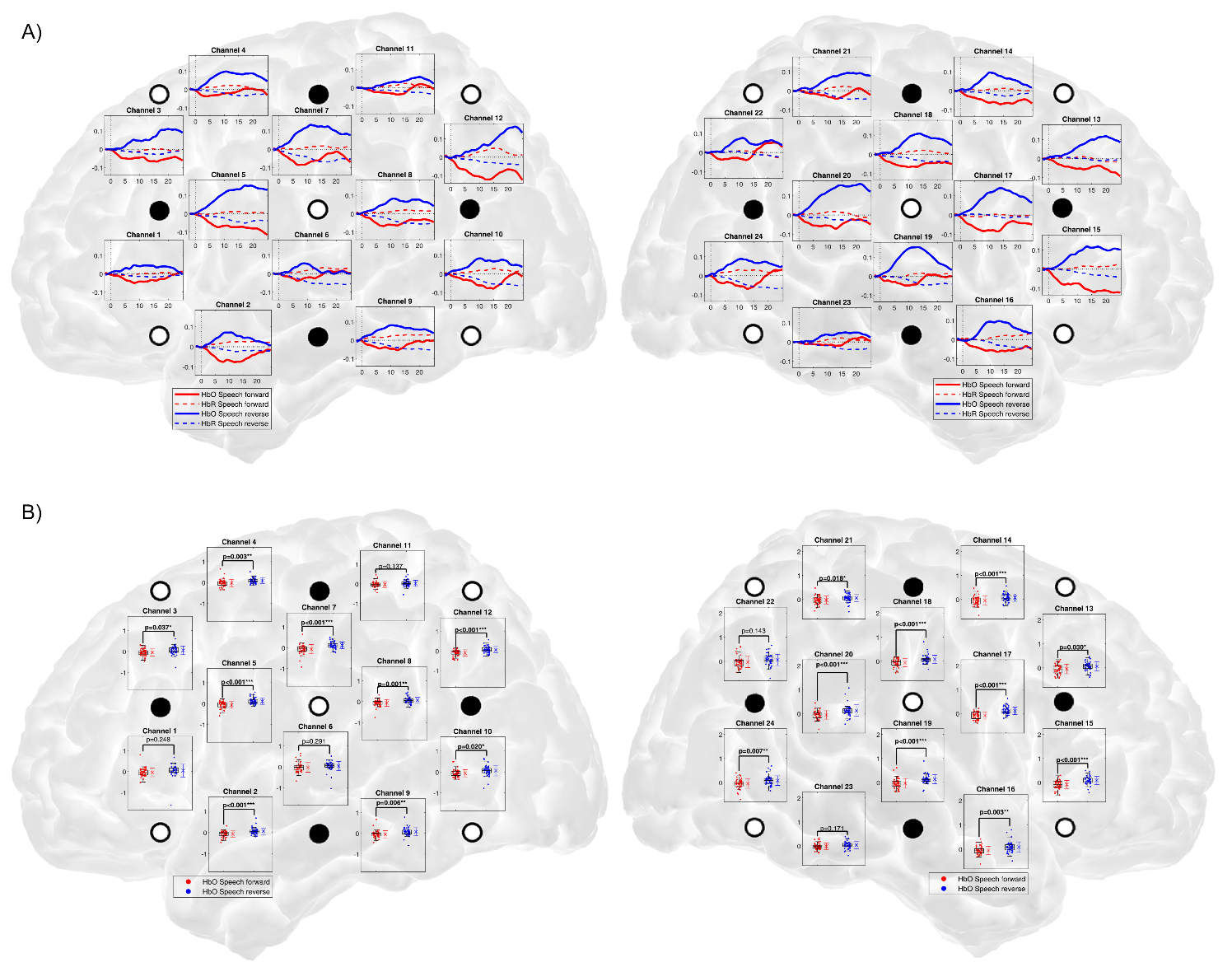
*Figure S2. Group_neg_ A) Mean time course of hemodynamic response for forward speech (red) and revese speech (blue) for each individual channel; B) channel-wise paired t-test comparison of mean HbO between conditions.

*Neonatal Speech Discrimination and Language Development Within Group_pos_ and Group_inv_*

Group_pos_ revealed a negative correlation between speech discrimination within the right hemisphere and phonological working memory indicating that less right hemisphere activation during speech discrimination is associated with better development of phonological memory ( r_s_ =-0.63, p = 0.005, Table S2). The use of morphological rules was furthermore negatively associated with right hemisphere involvement in speech discrimination, but the significance did not survive Bonferroni correction (r_s_ = -0.48, p =0.044). Group_inv_ showed a positive correlation between SPa_R_ and phonological short-term memory (r_s_ = 0.55, p = 0.012), indicating that a smaller negative difference between conditions in these two groups—meaning less involvement of the right hemisphere in speech discrimination—was significantly associated with better phonological working memory.

Table S2. Correlation of neonatal speech discrimination and language development within group_pos_ and group_inv_

|  | **Correlation with neonatal speech discrimination**  **r_s_ (p)** | | | | | |
| --- | --- | --- | --- | --- | --- | --- |
|  | **Study group with positive HbO values**  **(n = 22)** | | | **Study group with inverted HbO values**  **(n = 23)** | | |
|  | SPa | SPa_L_ | SPa_R_ | SPa | SPa_L_ | SPa_R_ |
| Language comprehension (z-score) | 0.04 (0.880) | -0.11 (0.670) | 0.12 (0.626) | 0.09 (0.702) | 0.17 (0.461) | 0.03 (0.908) |
| Sentence production (z-score) | -0.38 (0.115) | -0.33 (0.185) | -0.45 (0.060) | 0.07 (0.764) | 0.24 (0.319) | 0.11 (0.655) |
| Use of morphological rules (z-score) | -0.57 (0.563) | -0.1 (0.690) | **-0.48 (0.044)** | -0.37 (0.095) | 0.04 (0.861) | -0.28 (0.209) |
| Phonological short-term memory (z-score) | -0.16 (0.519) | 0.16 (0.538) | **-0.63 (0.005)*** | 0.33 (0.140) | 0.07 (0.760) | **0.55 (0.012)*** |

Note: SPa_L_, neural speech discrimination in left hemisphere; SPa_R_, neural speech discrimination in right hemisphere; LI_SP_, laterality index of neural speech discrimination; p < 0.05 is indicated in bold letters; * indicates significance after Bonferroni correction

In summary, using the algebraic difference in mean HbO changes between conditions within group_pos_ and group_inv_, instead of the absolute values within the whole group of participants, produced comparable results.

**Supplementary Note 2**

At the age of three years, children were invited for a follow-up investigation of their language abilities. In five children, tests had to be postponed due to three COVID lockdowns for more than one year, resulting in an age of five years at follow-up testing in these children. To rule out any bias of the age at test on study results, we here present the study findings of the subgroup of 3-year-old participants only.

The remaining 40 children (22 female) had a mean age of 3.55 years (SD 0.21, range 3.12 – 3.99). In these 40 children, language abilities were not associated with SES. Sentence production and use of morphological rules were associated with measures at birth, but these correlations did not survive corrections for multiple comparisons (Table S3). However, the group of preterm-born (n = 21) and term-born children (n = 19) significantly differed in their development of morphological rules (p = 0.044).

Table S3. Language test results at follow-up in 3-year-old participants (n = 40)

|  | **Mean (SD)**  **[range]** | **Correlation with measures at birth**  **r_s_ (p)** | | | | **Correlation with SES**  **r_s_ (p)** | **Preterm-born mean (SD) [range]** | **Term-born mean (SD)**  **[range]** | **Group differences**  **z (p)** |
| --- | --- | --- | --- | --- | --- | --- | --- | --- | --- |
|  |  | **Gestational age** | **Weight** | **Height** | **Head circum-ference** |  |  |  |  |
| Language comprehension  (z-score) | 0.28 (1.07)  [-2.10 – +2.10] | 0.29 (0.073) | 0.27 (0.100) | 0.23 (0.157) | 0.16 (0.313) | 0.23 (0.166) | 0.31 (1.03)  [-1.70 – +2.20] | 0.47 (1.15)  [-2.10 – +2.10] | 1.14 (0.258) |
| Sentence production (z-score) | 0.11 (1.05)  [-2.70 – +2.10] | **0.37 (0.025)** | **0.34 (0.042)** | 0.28 (0.099) | 0.18 (0.282) | 0.23 (0.185) | -0.21 (1.22)  [-2.70 – +2.10] | 0.42 (0.76)  [-1.20 – +2.00] | 1.75 (0.081) |
| Use of morphological rules  (z-score) | -0.29 (1.12)  [-2.10 – +1.70] | 0.30 (0.063) | **0.37 (0.021)** | **0.34 (0.031)** | **0.33 (0.037)** | 0.26 (0.112) | -0.25 (1.37)  [-2.10 – +2.89] | 0.11 (1.04)  [-2.00 – +1.70] | **2.01 (0.044)** |
| Phonological working memory  (z-score) | -0.17 (1.23)  [-2.89 – +2.50] | -0.03 (0.864) | 0.03 (0.874) | 0.01 (0.960) | -0.01 (0.978) | -0.03 (0.867) | 0.07 (1.26)  [-2.10 – +2.10] | -0.21 (1.23)  [-2.89 – +2.50] | 0.57 (0.573) |

Note: SES, socioeconomic background; p < 0.05 is indicated in bold letters

Findings of correlation analyses between speech discrimination at birth and language development in this subgroup of 3-year-olds were similar to the whole group of study participants. Neonatal speech discrimination measured over all channels (SP) did not significantly correlate with language development three to five years later (Table S4). In addition, neural speech discrimination within the left hemisphere was not significantly associated with language comprehension, language production, the use of morphological rules, or phonological short-term memory. However, neural speech discrimination within the right hemisphere was significantly negatively correlated with phonological short-term memory (r_s_ = -0.56, p < 0.001). Consequently, LI_SP_ significantly correlated with phonological short-term memory (r_s_ = 0.43, p = 0.006).

Overall, study findings in the subgroup of 3-year-old children point to a larger influence of prematurity at birth on language development compared to the whole study group. However, this subgroup analysis replicates the findings in the larger group that a more leftward speech discrimination in the neonates was significantly associated with better phonological working memory several years later.

Table S4. Correlation of neonatal speech discrimination and language outcome in 3-year-old participants (n = 40)

|  | **Correlation with neonatal speech discrimination**  **r_s_ (p)** | | | |
| --- | --- | --- | --- | --- |
|  | SP | SP_L_ | SP_R_ | LI_SP_ |
| Language comprehension (z-score) | -0.06 (0.703) | -0.12 (0.454) | 0.05 (0.783) | -0.11 (0.502) |
| Sentence production (z-score) | -0.24 (0.148) | -0.30 (0.069) | -0.25 (0.144) | -0.04 (0.812) |
| Use of morphological rules (z-score) | 0.11 (0.518) | -0.05 (0.752) | -0.04 (0.787) | -0.01 (0.936) |
| Phonological short-term memory (z-score) | -0.24 (0.139) | -0.06 (0.720) | **-0.56 (<0.001)*** | **0.43 (0.006)*** |

Note: SP, neural speech discrimination; SP_L_, neural speech discrimination in left hemisphere; SP_R_, neural speech discrimination in right hemisphere; LI_SP_, laterality index of neural speech discrimination; p < 0.05 is indicated in bold letters; * indicates significance after Bonferroni correction
